# Living Arrangements and Health-Related Quality of Life in Chinese Adolescents Who Migrate from Rural to Urban Schools: Mediating Effect of Social Support

**DOI:** 10.3390/ijerph14101249

**Published:** 2017-10-19

**Authors:** Haiyan Wu, Shan Wu, Haibo Wu, Qiming Xia, Ningxiu Li

**Affiliations:** 1 Department of Health-Related Social and Behavioral Science, West China School of Public Health, Sichuan University, Chengdu 610041, China; wuhaiyan1029@163.com; 2 School of Humanities and Management, Heilongjiang University of Chinese Medicine, Harbin 150040, China; 3 Medical Laboratory Technology, School of Clinical Medical, Ningxia Medical University, Yinchuan 750001, China; wushannxmu@163.com; 4 Touying Middle School, Guyuan 756000, China; tyzxwhb@126.com; 5 The Seventh Middle School, Guyuan 756000, China; xiaqiminggyqz@126.com

**Keywords:** rural-to-urban students, adolescents, living arrangements, quality of life, social support, mediating effect

## Abstract

Changes in living arrangements (from living with, or not living with family) may affect the health-related quality of life (HRQoL). This study aimed to investigate the impact of living arrangement on HRQoL among adolescents migrating from rural to urban schools, and whether social support, in addition to living with a family, had an impact. A cross-sectional survey of 459 school adolescents was carried out in two public schools in Guyuan County, Ningxia Hui Autonomous Region, China in 2015. The survey contained the following questionnaires: a self-designed questionnaire, the 12-item Short Form Health Survey (SF-12), and the Social Support Rating Scale (SSRS). Of the 459 adolescents sampled (aged 15.41 ± 1.07 years with range of 13 to 18), 61.7% were living with family, and 38.3% were not living with family. Those students not living with families had lower Mental Component Scale (MCS) scores as well as less social support overall. Those students, who were not living with families, also reported more chronic health problems and more alcohol consumption compared to those students living with families. Social support was a statistically significant mediating factor on the effect of living arrangements on MCS. Our findings demonstrated that those students, who were not living with families, tended to have more health-related quality of life issues, but social support partially mediated the relationship between living arrangements and health.

## 1. Introduction

China has experienced the most extensive internal migration in the last 30 years [1]. Millions (262 million) of farmers have moved from farmlands and agriculture areas into more urban areas and such migration includes 12.6 million rural-to-urban school-age children, who migrated with their parents [2]. The impact of the internal migration is far-reaching, particularly as the system of nine years of compulsory education has evolved dramatically during this period [3]. The launch of rural school closures and consolidations in 2001 has led to a decline of over 50% in the number of rural primary schools, with another reason being the low birth rate as a result of family planning policy [4]. For example, some rural schools relocated and moved to urban areas and became the main schools receiving migrated or transferred students. Rural children tend to migrate and become urban students, because the newly merged rural schools can be too far away and inconvenient to attend and, moreover, their parents tend to favor urban schools, which generally provide quality education [3,5,6]. For these reasons, some children with rural residency (Hukou) leave their rural homes and live in hostels in urban areas to attend urban school, without their parents. The urban schools, which enroll these students living without their families, receive large concentrations of migrant students.

Secondary education in China consists of three grades or years and is run by local governments and various business authorities. The 1986 “Compulsory Education Law” guaranteed at least nine years of education for all school-age children. In urban areas, quality schools—known as key schools—recruit students outside their school district to increase their income by charging fees [4]. The secondary school adolescents are entering puberty, a unique developmental period, where adolescents make important mental and physical growth. The living conditions and learning environment during this period can be vital for personal development [7], because they ensure and guarantee essential support for the adolescents from their parents and society [8]. The migrant students face challenges and particular pressures, compared with their non-migrant peers, in terms of fulfilling their daily needs, because both their living arrangements and the available social support change dramatically after migration.

Living conditions in childhood have profound short- and long-term effects on mental and physical development, even on future marriage [9]. Current studies on the effects of living arrangements on health mainly focus on children with divorced parents, or who are orphaned, children of political refugees, disabled children, and children in boarding schools [10,11,12,13,14,15]. Studies on migrant students mainly focus on the children who are left behind and/or comparisons with local children [16,17,18]. Migration is generally considered to have significant positive effects on migrant children’s material conditions, with no negative effects on their mental health [1].

Studies on the elderly show that the social support is a mediator in the relationship between health status and living conditions [19] and support from family and friends are consistently strong predictors of physical activity outcomes [20]. However, few studies have exploited the impact of living arrangements (living with, or not living, with parents) on the health of secondary school children moving from rural to urban areas. Adolescents facing stress might benefit more from family support compared to their peers without stressful life events and that friends may have a weaker presence in adolescent lives than expected [21].

Health-related quality of life is a multi-dimensional concept that includes physical, mental, and social domains. The concept of social support is similarly categorized and measured in different ways. These two concepts have some overlap; the current literature tends to agree that social relationships and social support improve physical and mental health, both directly and as stress buffers [22,23,24]. Therefore, we carried out the current study to investigate the relationship between the living arrangements and the health of the migrant rural children, as well as the role that social support plays. Our study hypothesized that the main effect would be in relation to the quality of life, whereby students living with their families would be in better health than those not living with families. The second goal of our study was to test whether social support mediates the effect of living arrangements on quality of life.

## 2. Materials and Methods

### 2.1. Subjects

The study was carried out among rural-to-urban adolescent students in Guyuan city from April to June 2015. The city of Guyuan is located in the southern mountainous area of Ningxia Hui Autonomous Region (as dry and windy) and the geographical central region of Xi’an, Yinchuan and Lanzhou metropolitan cities. Guyuan has a population of 1.22 million in 2010, and 44.38% of the population belongs to the Hui ethnic minority group—a Muslim minority who practice Islamic principles in culture, diet, and lifestyle [25]. Guyuan has eight middle schools, two are private schools with the highest education quality, and the rest are public schools. The listed in Table 1.

This cross-sectional survey used a two-stage cluster sampling method. Firstly, the Fifth and the Seventh Middle School of Guyuan were included, as most of their students were rural-to-urban students (students from the two schools accounted for 12% of all graduates in Guyuan, 2017). Secondly, five classes from grades 8–9 in each school were selected randomly, and all students in the selected classes were surveyed. A total of 500 questionnaires were distributed by head teachers who had been trained by the principal investigator; 459 valid questionnaires were gathered with a response rate of 91.8%. Fourteen questionnaires that were answered by students with a city residency (non-rural-to-urban immigrants) were not included in the final analysis.

### 2.2. Social-Demographical Characteristics

Information on the age, sex (boys or girls, ref. = boys), ethnicity (Han or Hui, ref. = Han), living arrangements (living with family, or not living with family, ref. = not living with family), and health-related behaviors including exercises, alcohol consuming were collected by the questionnaires. Examples of questions in this part were: “Do you have a chronic health problem?”; “Have you felt any discomfort during the past 14 days?”

### 2.3. Socioeconomic Status (SES)

The socioeconomic status of the adolescents was surveyed by collecting the educational level of the parents and the family income. The four education levels of parents in our study were: illiterate; primary school education; junior middle school education; and high school education or higher. The family income was represented by annual per capita income and including three levels: less than 2500 RMB; 2500–5500 RMB; and more than 5500 RMB; the tertile cutoffs were calculated based on the proportion of each level (equal population fell in each category).

### 2.4. Social Support

The Chinese version of Social Support Rating Scale (SSRS, developed by Shuiyuan Xiao) was used in this study [26]. The scale contains three constructs in 10 items: objective support (the degree of actual support an individual received in the past, three items), subjective support (the perceived interpersonal network that an individual can count on, four items), and availability of social support from family, friends and significant others (ultimate outcome of receiving support, three items). The scores for the scale range from 12 to 66, with higher scores indicating better social support [26]. The total social support is calculated by adding the three domains scores. The SSRS has been used in a wide range of studies on Chinese adolescents due to its high reliability and validity [27,28,29], with a two-month test-retest reliability of 0.92.

### 2.5. Quality of Life

Quality of life was measured by the 12-item Short Form Health Survey (SF-12) [30] (translated by Li et al in 2008 [31]), which is widely used for it is fast and easy to use, satisfactory reliability and validity [32]. The SF-12 Health Survey consists of 12 questions grouping into two components: a Physical Component Scale (PCS) and a Mental Component Scale (MCS). The two components of the SF-12 are appropriate indicators for health of Chinese adolescents [33,34]. The score of the SF-12 ranges from 0 to 100, with higher scores indicating better health, and a score of 50 or above indicates positive self-rated health, while a score below 50 indicates a negative self-rated health (Cronbach’s α = 0.87).

### 2.6. Data Analysis

**Figure 1:**
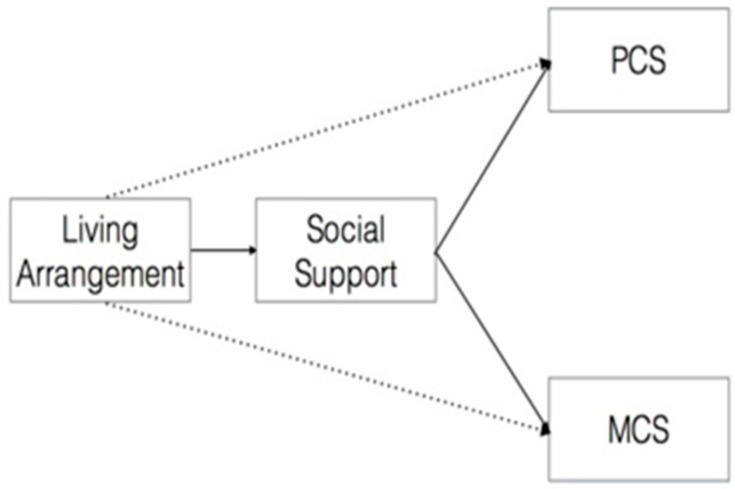
Mediation analysis model between living arrangement, social support, and quality of life in the school students. PCS: Physical Component Summary; MCS: Mental Component Summary. In this path for the mediator model, Living Arrangement = the independent variable, PCS/MCS = the dependent variable, Social Support = the mediating variable.

Data are expressed as percentages for counting data and mean ± standard deviation for continuous data. A chi-square test was used to examine differences in social demographic factors and risk behaviors between the two living arrangements. When the counts are small, we applied Fisher’s exact tests to analyze the chronic health problem, alcohol drinking, and regular exercise [35]. The student’s *t*-test was also used to compare quality of life and social support subscales score between children with different living arrangements. Cohen’s d was used as the measure of effect size. All statistical analyses were performed using IBM SPSS 22 software (IBM Corp., Chicago, IL, USA). PROCESS Procedure 2.16.3 was used for regression [36,37]. The number of bootstrap samples for bias corrected bootstrap confidence intervals is 5000, and the level of confidence for all confidence intervals in output is 95%. We used a simple mediation model to assess the effect of the rural-to-urban students’ living arrangements on quality of life both directly and indirectly through social support. This corresponds to the model depicted in Figure 1.

## 3. Results

### 3.1. Characteristics of Adolescent School Children by Living Arrangement

There were 459 school adolescents in the sample (aged 15.41 ± 1.07 years with range 13–18), 61.7% (*n* = 283) were living with family, and 38.3% (*n* = 176) were not living with family. In our preliminary analysis, age had no independent impact on the adolescents’ outcomes. The characteristics of the students’ living arrangements are summarized in Table 2. Approximately, 20% of the students were Hui ethnic people and 52% were boys. Nearly 14% reported that they had felt discomfort during the past 14 days. A significantly higher percentage of students, who were not living with families, had a chronic health problem compared to those students who were living with families (7.4% vs. 2.5%, X^2^ = 6.253, *p* = 0.017).

One third of the parents of the students had attained primary school education. The parents’ educational levels varied by sex; high school or higher education among fathers and mothers were 22% and 11% respectively, while rates of illiteracy were 9% and 26% respectively. There was a slightly higher annual per capita income, but lower parental educational level for students living with families, but the difference was not statistically significant. However, the prevalence of drinking alcohol was significant higher in students not living with families, than in students living with families (13% vs. 5%, X^2^ = 7.9, *p* = 0.005).

### 3.2. Living with Family Children Had Higher MCS Scores and Total Social Support

Students living with their families had higher HRQoL score comparing with students not living with their families and had statistically different scores on the MCS. The effect size of the difference was in the range of small to medium (48.74 vs. 45.10, *p* < 0.01, Cohen’s *d* = 0.37, Table 3). In addition, the migrant students who lived with their families had significantly higher levels of object social support (8.62 vs. 7.56, respectively, *p* < 0.01, Cohen’s *d* = 0.59) and significantly higher levels of global social support (37.58 vs. 36.65, respectively, *p* = 0.04, Cohen’s *d* = 0.20).

### 3.3. Social Support Mediation on Mental Health

The regression across the mediation models is summarized in Table 4. We did not perform regression on PCS because the impact of living arrangement on PCS was not significant. Global support was examined as a mediator. The mediation of global support on the effect of living arrangement was statistically significant in the MCS model (*R* = 0.23, *p* < 0.001, Table 4). Students who lived with families had a higher mental health score (total effects = 3.57, *p* < 0.001). However, global support significantly mediated the association between living arrangements and mental health. For mental health, the direct effects of living arrangement status were reduced (β = 3.31) after considering global support, but remained statistically significant (*p* < 0.001). Global support mediated 7% of the association between living arrangement and mental health, with indirect effects β = 0.27. The mediation effect was still statistically significant after controlled for sex and ethnicity (Table 4).

## 4. Discussion

This study examined disparities in HRQoL between adolescent students moving from rural to urban schools, who lived with families and those who did not live with families, but boarded in hostels and whether this connection could be mediated by social support. The results show that students not living with their families had lower MCS scores and this impact remained after ethnicity and sex were controlled for. Social support mediated the impact of living arrangement on MCS, indicating that students who were not living with families had poorer MCS, attributable to the lack of available social support.

Previous studies have demonstrated the importance of social factors for migrant adolescents [17]. Teacher support can partially mediate the relationship between perceived discrimination and antisocial behaviors of rural-to-urban migrant adolescents [38]. Migration and social support served as important factors explaining mental health outcomes; migrating alone and receiving little social support was associated with the worse mental health outcomes (depression, anxiety, and mental distress) [39]. Immigrant adolescents have reported higher levels of psychological distress and lower social support than their non-immigrant peers [40].

This study provides valuable information involving the health status of adolescents who migrate from rural to urban schools. Both the students and their families need to understand what kind of social support band how families can provide social support during school years. Adolescents, who are not living with families while they are at school, should be consulted about the different types of social support they need. Specific strategies are required to ensure that adolescents, who are not living with their families, have people from whom they can ask for help. Though social needs may vary greatly among those not living with families, social supports can be provided by a range of sources liketeachers, friends, and class teammates [23,41]. A previous study, which examined support-seeking behavior in migrant adolescents, showed that the adolescents relied on teachers and classmates as a source of social support. Adolescents are not fully socialized; peers have the greatest impact on their life when they are not living with family, indicating that parents and teachers should pay attention to the child’s friends and supervise the child's behaviors [18,23].

The impact of living arrangement is the prime focus of this study, and a subsequent study should continue to explore why living apart from families has such a great impact on the health of the children. Although the mediating effect is significant, questions remain about what social support has the greatest impact and what role social support plays. The answers to these questions are still not clear and need further investigation. Obviously, there are other types of mediators influencing the effect of living arrangement on MCS. Parental expectations could be another mediator [23]. Adolescents, who are not living with their families, may be under greater learning pressures, because their parents while allowing them to live alone in the city, may expect higher academic performance. Moreover, adolescents who are not living with families, may need more time to take care of life chores and have greater economic burdens. Comparison in the mental health of rural and urban students not living with families, should be conducted in the future as adolescents, who do not live with their families, are partly deprived of important experiences.

In our study, health-related behavioral problems and a higher incidence of chronic disease were found in adolescents not living with families, which is consistent with previous studies [42,43]. For example, one study showed that young children who lived with family members had better growth measurements (a long-term health indicator) than their peers living in institutional settings [44]; students living at home practised more sports and more frequently consumed raw and cooked vegetables, fish, meat and poultry, fresh fruit, eggs, bread/cereals, and hence were in better health conditions [45]. Conversely, students living away from home consumed more packaged/ready food, beer and spirits, milk and chips. The majority of students living alone reported a modification of dietary habits since they left their families. Furthermore, they appeared to be more likely to gain weight to a greater extent than students living with family [45]. Living with their family can fulfill an adolescent’s emotional and feeling needs too.

A significant difference was found on adolescents’ mental health, but not physical health, between those living with families and those not living with families. Adolescents are generally healthy [46] and while living arrangements may not have an immediate effect on physical health, they can be associated with negative consequences on mental health. Mental health is more sensitive than physical health. In this study, those who did not live with families had more chronic health problem, but significantly poorer in physical health scores.

This study has limitations. The nature of cross-sectional study, the small sample size, together with other restrictions in sampling (schools and areas) limited the generalization of the results. However, because the students surveyed were in the second or third year of middle school (transfers from another school were rare), and they had generally lived in the city for at least a year and a half at the time of surveying and by then had largely accommodated to city life, which could compensate for the disadvantages a cross-sectional study. Another limitation may derive from relying on self-report questionnaires and as researchers were not in the classrooms at the time of data collection, there may be bias in the results based on teacher influence. Finally, we did not collect data to explain why the students, who did not live with family, lived away from them families. Possible explanations such as financial issues, complications from divorce, etc., could be confounding stressors that could have major impacts on students’ quality of life.

## 5. Conclusions

In conclusion, our study in Guyuan of secondary school adolescents showed the impact of living arrangements on the health of students, who have migrated from rural to urban areas. Those students who are not living with their families tend to have more HRQoL issues. The impact of living arrangements on health is mediated by social support, suggesting that more social support from schools should be provided to urban migrant students. Furthermore, the findings may inform the development of mental health services and programmers that can potentially benefit a large number of internal migrant adolescents in China.

## Figures and Tables

**Table 1:** Characteristics of the eight middle schools in Guyuan, Ningxia.

Name	Characteristics	Number and Proportion of Graduates in 2017
Wuyuan middle school	Private school, key school, since 1941	890 (17.3%)
Hongwen middle school	Private school, key school, since 1973	814 (15.80%)
The Third middle school	Public school, since 1979	1043 (20.20%)
Muslim Secondary school	Public school, more than half of the students are Hui ethnic group, since 1984	325 (6.30%)
The Fourth middle school	Public school, since 1985	463 (9.00%)
The Fifth middle school	public school, rebuilt and relocated under the policy of closures and consolidations in 2002 ^1^	281 (5.5%)
The Sixth middle school	Public school, the newest school for Urbanization with increased number of students, since 2003	1017 (19.7%)
The Seventh middle school	Public school, rebuilt and relocated under the policy of closures and consolidations in 2006 ^2^	322 (6.25%)

^1^ Rebuilt with the resources from rural schools (Nanjiao School and Xijiao’s Second school); ^2^ Rebuilt with the resources from rural schools (Shili School and Chenershan School).

**Table 2:** Characteristics of students surveyed according to living arrangements.

Variables	Total (*n* = 459)		Living with Family (*n* = 283)		Not Living with Family (*n* = 176)		X^2^	*p*
	*n*	%	*n*	%	*n*	%		
Control variables								
Ethnic groups							0.111	0.815
Han	361	78.6	224	79.2	137	77.8		
Hui	98	21.4	59	20.8	39	22.2		
Sex							0.877	0.388
male	237	51.6	151	53.4	86	48.9		
female	222	48.4	132	46.6	90	51.1		
Have you felt uncomfortable at any time during the last 14 days							1.409	0.262
yes	62	13.5	34	12.0	28	15.9		
No	397	86.5	249	88.0	148	84.1		
Do you have a chronic health problem							6.253	0.017
Yes	22	4.8	7	2.5	13	7.4		
No	437	95.2	272	97.5	161	91.5		
SES								
Father’s educational level							1.546	0.672
Illiterate	41	8.9	24	8.8	17	10.0		
Primary school	152	33.1	94	34.3	58	34.1		
Junior middle school	149	33.6	97	35.4	52	30.6		
High school or higher	102	22.2	59	21.5	43	25.3		
Mother’s educational level							2.856	0.416
Illiterate	117	25.5	76	27.6	41	24.3		
Primary school	153	33.3	87	31.6	66	39.1		
Junior middle school	125	27.2	82	29.8	43	25.4		
High school or higher	49	10.7	30	10.9	19	11.2		
Annual per capita income							3.239	0.198
<2500	161	37.4	108	40.6	53	32.1		
2500–5500	143	33.2	85	32	58	35.2		
>5500	127	29.5	73	27.4	54	32.7		
Health-related behavior								
Drinking alcohol							7.9	0.005
No	350	92.1	230	95.0	120	87.0		
Yes	30	7.9	12	5.0	18	13.0		
Regular exercise							0.01	0.991
Yes	349	91.8	217	91.9	132	91.7		
No	31	8.2	19	8.1	12	8.3		

**Table 3:** Descriptive statistics for HRQoL variables, social support by living arrangements.

Variables	Total		Living with Family (*n* = 283)		Not Living with Family (*n* = 176)		*t*	*p*	Cohen‘s *d*
	Mean	SD	Mean	SD	Mean	SD			
PCS	51.61	7.13	51.81	6.59	51.29	7.93	−0.727	0.47	0.07
MCS	47.35	9.75	48.74	8.97	45.10	10.53	−3.807	0.00	0.37
Objective Support	8.21	1.89	8.62	1.80	7.56	1.84	−6.112	0.00	0.59
Subjective Support	21.00	2.78	20.92	2.75	21.14	2.83	0.829	0.41	0.08
Availability Support	8.01	1.86	8.04	1.88	7.96	1.83	−0.460	0.65	0.04
Global Support	37.22	4.63	37.58	4.52	36.65	4.77	−2.091	0.04	0.20

**Table 4:** Mediation effect of social support in this study.

**Unadjusted**	**Effect**	**SE**	***t***	***p***	**LLCI**	**ULCI**
Total effect	3.5753	0.9225	3.8758	0.0001	1.7625	5.3881
Direct Effect	3.3096	0.9184	3.6039	0.0003	1.5049	5.1144
Indirect effect	Effect	bootSE			bootLLCI	bootULCI
Global support	0.2657	0.1598			0.028	0.6902
Adjusted (by sex and ethnicity)	Effect	SE	*t*	*p*	LLCI	ULCI
Total effect	3.4931	0.9225	3.8758	0.0001	1.7625	5.3881
Direct Effect	3.2175	0.9174	3.5071	0.0005	1.4145	5.0205
Indirect effect	Effect	bootSE			bootLLCI	bootULCI
Global support	0.2756	0.1624			0.0365	0.6968
